# Feasibility and candidate risk factors for postoperative delirium in older ward patients: a prospective pilot study

**DOI:** 10.1186/s12871-026-04321-w

**Published:** 2026-10-06

**Authors:** Shaymaa Al-Dabooni, F. Wappler, J. Weber, T. Annecke, M. Fritz, R. Lefering, I. Feinkohl, J. Defosse

**Affiliations:** 1 Department of Anesthesiology and Intensive Care Medicine, Cologne- Merheim Medical Center, University of Witten-Herdecke, Cologne, Germany; 2 Institute for Research in Operative Medicine (IFOM), University of Witten- Herdecke, Cologne, Germany; 3 Epidemiology Research Group, Faculty of Health Sciences Brandenburg, University of Potsdam, Potsdam, Germany; 4 Molecular Epidemiology Research Group, Max Delbrück Center for Molecular Medicine in the Helmholtz Association (MDC), Berlin, Germany

**Keywords:** Delirium, Frailty, Cognitive impairment, Malnutrition, Handgrip strength

## Abstract

**Background:**

Postoperative delirium (POD) is a frequent neuropsychiatric complication after surgery, characterized by acute disturbances in attention, awareness, and cognition. This pilot study assessed the feasibility of a multidimensional assessment protocol and investigated candidate risk factors for POD in older patients undergoing elective surgery.

**Methods:**

This prospective, observational, non-interventional, single-center pilot study enrolled 150 patients aged ≥ 65 years undergoing elective surgery lasting ≥ 60 min. Patients with dementia, neurological disorders (documented diagnoses), or emergency procedures were excluded. After enrollment, 10 patients were excluded due to surgery postponement or unplanned ICU admission, leaving 140 patients for analysis. Data were collected preoperatively, on postoperative days 1–3. Intraoperative and laboratory data were obtained from databases. Handgrip strength was measured by dynamometry. POD was assessed using the CAM-ICU once daily on postoperative days 1–3 by a single trained investigator. Cognitive function and reserve were evaluated using MMSE and MWT-B, frailty with the Clinical Frailty Scale, nutritional status with screening tools, and pain via the Numeric Rating Scale. Descriptive and inferential statistics were applied.

**Results:**

POD incidence was 5.0% (n = 7/140; 95% CI: 2.0%–10.0%). Frailty (n = 4/7 [57%] vs. n = 26/133 [20%]) and malnutrition (n = 4/7 [57%] vs. n = 29/133 [22%]) were more common in the delirium group. In an exploratory, unpowered male subgroup analysis, patients with POD had lower preoperative handgrip strength; no statistically meaningful difference was testable in women (n = 3 with POD). During anesthesia induction, patients with POD experienced more hypotensive episodes (MAP<55 mmHg), (n = 3/7 [43%] vs. n = 11/133 [8.0%]). No comparison remained significant after Holm-Bonferroni correction.

**Conclusion:**

This pilot study demonstrates the feasibility of prospective POD assessment on a general surgical ward and provides an incidence estimate (5.0%) to inform sample-size calculation for confirmatory trials. Frailty, lower preoperative handgrip strength in men, and induction hypotension were more frequent among patients with POD. Given the low event rate and multiple comparisons, these associations are hypothesis-generating and require validation in adequately powered studies.

**Trial registration:**

German Clinical Trials Register DRKS00031157. Registered 20.03.2023.

## Background

Postoperative delirium (POD) is an acute neuropsychiatric complication characterized by disturbances in attention, awareness, and cognition. It typically occurs in the immediate postoperative period resulting from transient dysfunction of cerebral networks. POD presents with fluctuating levels of consciousness, disorientation, and impaired memory, posing significant risks for adverse outcomes. Duration ranges from two to five days [1–3].

Subtypes include hyperactive, hypoactive, and mixed forms [4]. Delirium is associated with adverse clinical outcomes, including increased mortality, prolonged hospital stay, and healthcare costs [4].

The vulnerability-threshold model highlights the interplay between predisposing factors (advanced age, cognitive impairment, malnutrition) and precipitating factors (major surgery, infection). Cerebral hypoxia may occur in the context of perioperative cerebral hypoperfusion and may contribute to cognitive dysfunction [5–7]. Postoperative pain is an independent risk factor for delirium [8]. Cognitive reserve, including education and baseline intellectual function, influences susceptibility to POD; however, evidence remains limited by small sample sizes and heterogeneous populations [9].

Validated tools are used to standardize delirium assessment. The Confusion Assessment Method for the Intensive Care Unit (CAM-ICU) is a validated and reliable tool for delirium assessment in mechanically ventilated ICU patients [10]. Regarding therapy, management focuses on monitoring, pain control, and correction of physiological imbalances [5]. Pharmacological options remain supportive [11].

This pilot study aimed to identify candidate risk factors for POD in older patients undergoing elective surgery on general wards. Specifically, it examined whether cognitive function, frailty, nutritional status, and perioperative hemodynamics are associated with POD. The primary objective is to inform hypothesis-driven study design and sample size calculation for a future confirmatory trial.

## Methods

### Study population

This non-interventional, single-center pilot study was conducted between May 22, 2023, and December 22, 2024, at Cologne-Merheim Medical Center, University of Witten/Herdecke. Approved by the local ethics committee (Ref. 15/2023) and registered (DRKS00031157). A total of *n* = 150 patients were enrolled. Eligible participants were aged ≥ 65 years, scheduled for elective surgery ≥ 60 min, and not expected to require ICU admission. Exclusion criteria: diagnosed dementia or neurological disorders, acute central nervous system disease, sepsis, intoxication, intracranial surgery, hospital stay < 3 days, insufficient language proficiency, deafness, and emergency procedures. Preoperative cognitive function was assessed using the Mini-Mental State Examination (MMSE). An MMSE score of ≤ 23 was considered indicative of mild cognitive impairment (MCI). Participation was voluntary.

### Data collection

Data were collected during a preoperative baseline visit and on postoperative days 1 to 3 (D1–D3). Long-term follow-up on postoperative day 90 (D90) was planned but was not evaluated in the final analysis. Overall, 103 of 140 patients (73.6%) were successfully contacted by telephone at D90, including 102 of 133 patients without POD and 1 of 7 patients who developed POD. Given the substantial differential loss to follow-up in the POD group, the D90 endpoint was excluded from the final analysis.

Cognitive function was evaluated using the MMSE [12]; premorbid cognitive reserve was assessed using the MWT-B. POD was screened once daily (between 14:00 and 16:00) from D1 to D3 using the CAM-ICU by a single, specifically trained investigator. POD assessment was performed in all 140 patients on D1. On D2, 112/140 patients (80%) remained available for assessment, and on D3, 88/140 patients (63%) were assessed. Missed assessments on D2 and D3 were due to hospital discharge; no patient declined assessment. All seven patients who developed POD completed CAM-ICU assessments on D1–D3. Patients discharged before completion of the three-day assessment period who had no positive CAM-ICU assessment during the available in-hospital observation period were classified as without POD; assessments after hospital discharge were not performed. Handgrip strength was measured using a digital dynamometer (Smedley Hand Dynamometer) preoperatively and daily postoperatively; measurements were executed in a seated position prior to analgesic administration. Frailty was evaluated preoperatively using the Clinical Frailty Scale (CFS), with frailty defined categorically as a score ≥ 5 [13]. Nutritional risk was assessed using the Nutritional Risk Screening 2002 (NRS 2002), with malnutrition defined as a total score ≥ 3 [14]. Pain was measured using the Numeric Rating Scale (NRS). Complications were graded according to the Dindo–Clavien classification [15]. Biomarkers were not collected.

Clinical data were extracted from the electronic anesthesia documentation system (Medlinq). Hemodynamic parameters were obtained from routine intraoperative non-invasive blood pressure monitoring (3–5 min intervals). Hypotension was defined as mean arterial pressure (MAP) < 55 mmHg. Percentage blood pressure deviation was calculated as: [(pre-induction resting Baseline MAP − intraoperative MAP) / Baseline MAP × 100].

### Sample size calculation

Required sample size was calculated based on an expected POD incidence of 10%, a 95% confidence level (Z = 1.96), and precision of ± 5%, yielding a minimum of *n* = 140 patients. Target sample size was increased to *n* = 150 to compensate for protocol deviations. The calculation was designed strictly to estimate incidence, not to detect differences between subgroups.

### Statistical analysis

Data were analyzed using descriptive and inferential statistics. Continuous variables are presented as medians with interquartile ranges [IQR]. Categorical variables are reported as counts and percentages with absolute risk differences. Given the restricted number of events (*n* = 7), multivariable adjustment was not statistically feasible. After Holm-Bonferroni correction, none of the 27 unadjusted comparisons in Table 1 remained statistically significant; all reported p-values are therefore unadjusted and strictly hypothesis-generating. Analyses were conducted with IBM SPSS Statistics version 29.0.

**Table 1:** Perioperative Characteristics Stratified by Postoperative Delirium Status

Variable	Without Delirium (*n* = 133)	With Delirium (*n* = 7)	Absolute Difference	*P*-value
Age, years	75.0 [68.0–79.0]	81.0 [78.0–85.0]	+ 6.0 years	0.067
Sex (Male)	67/133 (50%)	4/7 (57%)	+ 7pp	1.000
BMI (kg/m²)	26.0 [24.0–29.0]	26.0 [26.0–27.5]	—	0.490
Smoking	23/133 (17%)	1/7 (14%)	-3pp	1.000
Frailty (CFS ≥ 5)	26/133 (20%)	4/7 (57%)	+ 38pp	0.038
Malnutrition (NRS 2002 ≥ 3)	29/133 (22%)	4/7 (57%)	+ 35pp	0.053
MWT-B Score	30.0 [26.0–33.0]	26.0 [20.0–34.0]	-4.0	0.437
MMSE Score	29.0 [27.0–30.0]	28.0 [25.0–29.0]	-1.0	0.300
Preoperative handgrip strength, male (kg)	34.0 [29.0–40.0] (*n* = 67)	23.5 [18.5–26.5] (*n* = 4)	-10.5 kg	0.012
Preoperative handgrip strength, female (kg)	21.0 [16.0–25.0] (*n* = 66)	25.0 [23.5–25.5] (*n* = 3)	+ 4.0 kg	—
Hypertension	81/133 (61%)	4/7 (57%)	-4pp	1.000
Diabetes mellitus	22/133 (17%)	1/7 (14%)	-2pp	1.000
Hemoglobin (g/dL)	13.5 [12.2–14.6]	12.3 [11.5–13.8]	—	0.300
Leukocytes (/nl)	7.1 [5.8–8.8]	5.3 [4.8–7.5]	—	0.060
Sodium (mmol/L)	139.0 [136.0–141.0]	139.0 [138.0–140.0]	—	0.320
SpO₂ (%)	97.0 [95.0–98.0]	97.0 [96.0–98.0]	—	0.910
Diuretics	32/130 (25%)	2/7 (29%)	+ 4pp	1.000
Statins	43/132 (33%)	2/7 (29%)	-4pp	1.000
ACE inhibitors	63/133 (47%)	2/7 (29%)	-19pp	0.450
Calcium channel blockers	36/133 (27%)	2/7 (29%)	+ 2pp	1.000
Beta blockers	39/133 (29%)	3/7 (43%)	+ 14pp	0.428
Oral opioids	16/133 (12%)	1/7 (14%)	+ 2pp	1.000
ASS	27/133 (20%)	2/7 (29%)	+ 8pp	0.634
Patients with ≥ 1 hypotensive episode during anesthesia induction (< 55 mmHg)	11/133 (8.0%)	3/7 (42.9%)	+ 35pp	0.022
Patients with ≥ 1 intraoperative hypotensive episode (< 55 mmHg)	20/133 (15.0%)	0/7 (0.0%)	-15pp	0.593
Duration of hypotensive episodes during anesthesia induction (< 55 mmHg) (median [IQR])	2.68 [2.21–3.78] (*n* = 11)	3.97 [2.12–6.50] (*n* = 3)	—	0.659
Percentage deviations in blood pressure during anesthesia induction	29.8 [21.0–40.2]	42.2 [31.5–47.5]	—	0.340
Percentage intraoperative blood pressure deviations	31.2 [21.5–43.8]	40.0 [32.0–48.5]	—	0.200

## Results

**Fig. 1:**
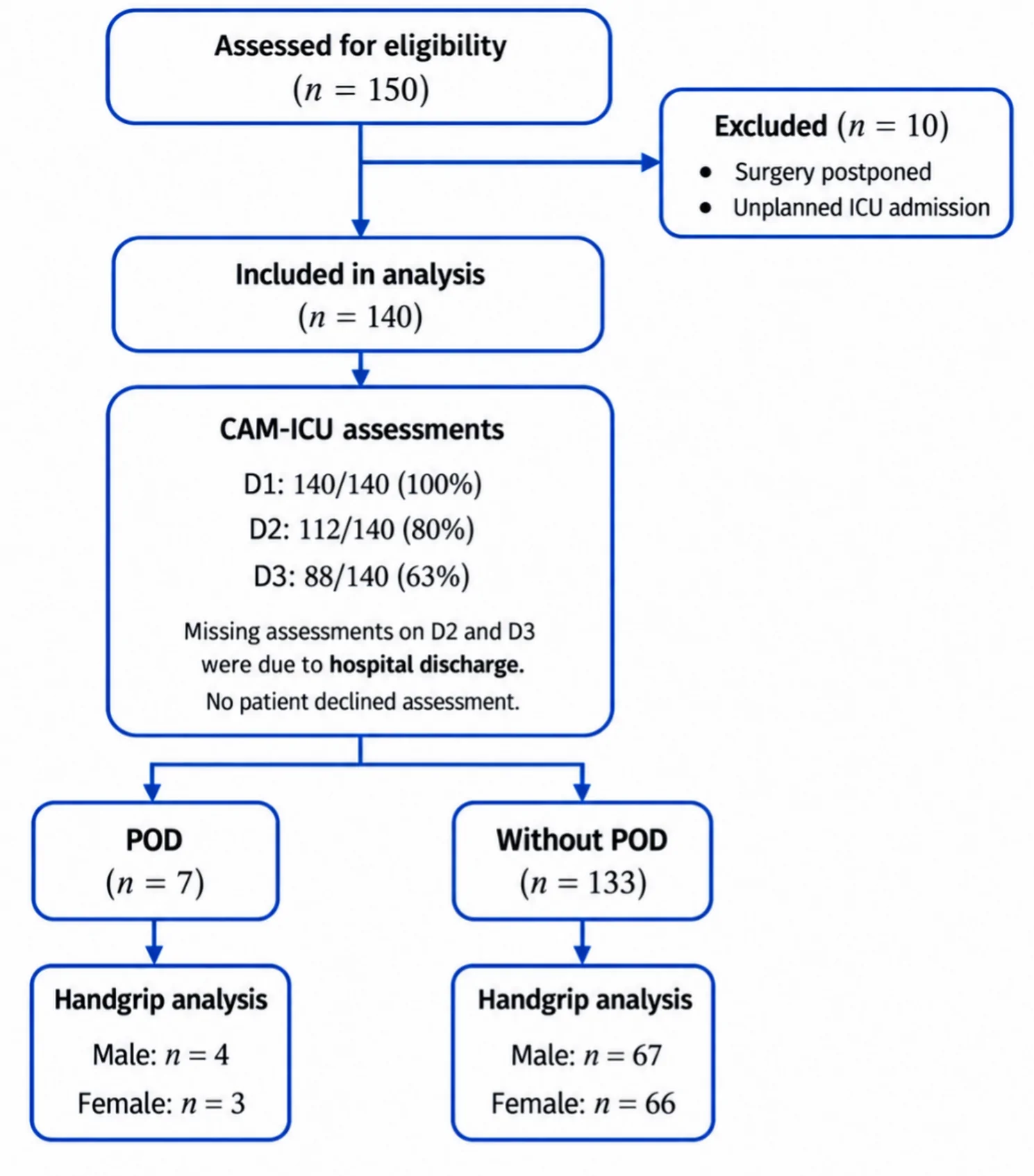
STROBE participant flow diagram illustrating patient enrollment, exclusions, postoperative CAM-ICU assessments, the final study cohort, and sex-specific handgrip analysis subgroups

A total of *n* = 150 patients were enrolled. *n* = 10 patients were excluded due to surgery postponement or unplanned ICU admission, leaving *n* = 140 patients for final analysis (Fig. 1). Overall POD incidence was 5.0% (*n* = 7/140; 95% CI: 2.0%–10.0%).

### Primary outcome

Frailty was more common among patients with POD (*n* = 4/7 [57%] vs. *n* = 26/133 [20%]; Fisher’s *p* = 0.038). In an exploratory, male-only subgroup analysis, preoperative handgrip strength was lower in the POD group (*n* = 4, 23.5 [18.5–26.5] vs. *n* = 67, 34.0 [29.0–40.0] kg; MWU *p* = 0.012). In female patients, no statistically meaningful difference was testable (*n* = 3 with POD).

Malnutrition was more common in the POD group (*n* = 4/7 [57%] vs. *n* = 29/133 [22%]; Fisher’s *p* = 0.053), although this difference did not reach statistical significance.

Age did not reach statistical significance between cohorts (81.0 [78.0–85.0] vs. 75.0 [68.0–79.0] years; MWU *p* = 0.067). Similarly, preoperative cognitive reserve was comparable between the POD group and the non-POD group (MWT-B: 26.0 [20.0–34.0] vs. 30.0 [26.0–33.0]; MWU *p* = 0.437).

### Intraoperative factors

During anesthesia induction, patients who developed POD exhibited a higher frequency of hypotensive episodes (*n* = 3/7 [43%] vs. *n* = 11/133 [8.0%]; Fisher’s *p* = 0.022) and a comparable median duration (3.97 [2.12–6.50] vs. 2.68 [2.21–3.78] minutes; MWU *p* = 0.659). In contrast to induction, no patient who developed POD experienced an intraoperative hypotensive episode, whereas such episodes were observed in *n* = 20/133 (15.0%) patients without POD.

Other variables, including sex distribution (*n* = 4/7 male [57%] vs. *n* = 67/133 male [50%]; *p* = 1.000), BMI (26.0 [26.0–27.5] vs. 26.0 [24.0–29.0] kg/m²; *p* = 0.490), smoking status, comorbidities, preoperative laboratory parameters, and percentage blood pressure deviations, were comparable between groups. The complete perioperative and postoperative characteristics stratified by postoperative delirium status are presented in Table 1.

Continuous variables are presented as medians with interquartile ranges [IQR]. Denominators vary because of missing medication data: data were missing for three patients for diuretics and one patient for statins. The female handgrip difference (+ 4.0 kg) is descriptive only; no statistical comparison was performed (*n* = 3 with POD). Abbreviations: ASS: Acetylsalicylic Acid; BMI: Body Mass Index; CFS: Clinical Frailty Scale; IQR: Interquartile Range; MAP: Mean Arterial Pressure; MMSE: Mini-Mental State Examination; MWU: Mann-Whitney U test; MWT-B: Mehrfachwahl-Wortschatz-Intelligenztest Version B; *n*: Number of patients; NRS 2002: Nutritional Risk Screening 2002; POD: Postoperative Delirium; pp: percentage points; SpO₂: Peripheral capillary oxygen saturation.

## Discussion

This prospective pilot study investigated associations between perioperative factors and POD development. Observed POD incidence was 5.0%, aligning with recent structured screening reports [16–18].

Detection bias represents a leading explanation for this low incidence rate. Three ascertainment features likely underestimated true POD. First, the CAM-ICU is validated for mechanically ventilated ICU patients; its sensitivity in communicative ward patients is less established than tools like the 3D-CAM or 4AT, potentially under-detecting hypoactive delirium. Second, assessment was restricted to once daily on postoperative days 1–3, rendering fluctuating or nocturnal episodes susceptible to omission. Third, patients discharged before completion of the three-day assessment period without a positive CAM-ICU assessment were classified as without POD, which may have further contributed to underestimation of the true POD incidence.

Preoperative frailty was more frequent in the POD cohort, consistent with previous evidence demonstrating an association between preoperative frailty and POD [19, 20]. Furthermore, previous studies have reported an association between malnutrition (identified via NRS 2002) and POD [21–24], potentially mediated by systemic inflammatory pathways [25]. However, this association did not reach statistical significance in the present cohort. Likewise, the association between premorbid cognitive reserve (MWT-B) and POD was not statistically significant. Although previous literature suggests that higher cognitive capacity may confer resilience against cognitive decline [9, 26, 27], this relationship could not be confirmed in the present study. These findings may reflect the limited sample size and the resulting reduced statistical power.

Male-specific analysis of preoperative handgrip strength demonstrated lower absolute strength in the POD cohort. No statistically meaningful difference was testable in women due to the very limited number of events (*n* = 3 with POD). Diminished muscle strength serves as an indirect marker of physical vulnerability closely tied to frailty [28–30]. Importantly, following Holm–Bonferroni correction for multiple comparisons, no individual metrics retained statistical significance.

A higher frequency of hypotensive episodes during anesthesia induction was observed in POD patients. Low blood pressure during induction may compromise cerebral perfusion [31–33]. However, in direct contrast to induction, none of the patients who developed POD experienced an intraoperative hypotensive episode, compared to 15.0% of non-POD patients. This zero, direction-inverse finding contradicts a simple dose-response cerebral hypoperfusion mechanism in this cohort, suggesting that either closer intraoperative management mitigated risk, or the finding reflects the small event scale.

### Methodological limitations

The absence of broad statistical significance across variables reflects core limitations. As a single-center pilot with seven events, statistical power is restricted. Given the small event rate (*n* = 7) and the high number of unadjusted tests, no comparison survived Holm-Bonferroni correction for multiplicity. The identified associations must therefore be interpreted exclusively as hypothesis-generating signals rather than established predictors. Additionally, four of 140 patients (2.9%) classified as having MCI had an MMSE score ≤ 23; none developed POD. Consequently, undiagnosed dementia could not be excluded as a potential confounder. Finally, duration tracking for hypotensive episodes relied on intermittent 3–5 min sampling limits. With median episode durations of ~ 4 min intersecting the resolution limit of the monitoring method, duration estimates are highly susceptible to artifact and should be interpreted strictly as approximations.

## Conclusions

This pilot demonstrates the feasibility of prospective, multidimensional POD assessment on a general ward and provides a precision-based incidence estimate (5.0%, 95% CI 2.0–10.0%) to inform sample-size calculations for future confirmatory trials. Frailty, lower preoperative handgrip strength in men, and induction hypotension were observed more frequently among patients developing POD in unadjusted comparisons. Given the low event rate, the number of comparisons performed, and the fact that no comparison survived correction for multiplicity, these associations represent hypotheses for confirmatory testing rather than established predictors. Whether targeted interventions aimed at improving muscle strength and physical resilience, as well as maintaining intraoperative hemodynamic stability, ultimately reduce POD risk requires validation in adequately powered, large-scale cohorts.

## Data Availability

The datasets used and/or analysed during the current study are available from the corresponding author on reasonable request.

## Abbreviations

BMI: Body Mass Index
CAM-ICU: Confusion Assessment Method for the Intensive Care Unit
CFS: Clinical Frailty Scale
CI: Confidence Interval
D1: Postoperative Day 1
D2: Postoperative Day 2
D3: Postoperative Day 3
D90: Postoperative Day 90
IQR: Interquartile Range
MAP: Mean Arterial Pressure
MCI: Mild Cognitive Impairment
MMSE: Mini-Mental State Examination
MWT-B: Mehrfachwahl-Wortschatz-Intelligenztest Version B
MWU: Mann-Whitney U test
NRS: Numeric Rating Scale
NRS 2002: Nutritional Risk Screening 2002
POD: Postoperative Delirium
STROBE: Strengthening the Reporting of Observational Studies in Epidemiology
